# ﻿*Loniceralanzhouensis* (Caprifoliaceae), a new species from Gansu, Northwest China

**DOI:** 10.3897/phytokeys.256.142365

**Published:** 2025-05-19

**Authors:** Shun Liu, Yu-Jin Wang

**Affiliations:** 1 State Key Laboratory of Herbage Improvement and Grassland Agro-ecosystems, College of Ecology, Lanzhou University, No 222, Tian-Shui-South Road, Lanzhou University, Lanzhou 730000, China Lanzhou University Lanzhou China

**Keywords:** *Loniceralanzhouensis*, molecular phylogeny, new species, taxonomy

## Abstract

*Loniceralanzhouensis* (Caprifoliaceae), a new species from Lanzhou City, Gansu province, China, is described and illustrated. The new species is morphologically similar to *L.webbiana* in that it has a bilabiate corolla and a long peduncle. It differs by narrowly lanceolate leaves (vs. ovate to ovate-lanceolate), yellow-green corolla at the beginning and reddish-purple when mature (vs. purple-red) and long involucral bracts (vs. short). The new species is supported by the genetic differentiation and phylogenetic analysis based on ITS and the combined sequences of chloroplast *psb*A-*trn*H and *mat*K fragments.

## ﻿Introduction

The genus *Lonicera*, the second largest genus within the family Caprifoliaceae, is characterized by the combination of leaves opposite, cymes opposite and usually reduced to paired flowers, corolla 5-lobed, stamens 5, berry red, black or green. About 180 species were recognized and distributed in North Africa, Asia, Europe and North America, of which 57 can be found in China (Yang et al. 2011). Hsu and Wang (1988) proposed a system for Chinese species, largely following the world-wide monograph of Rehder (1903), by splitting the genus into two subgenera, i.e., *Lonicera* and *Chamaecerasus*. Subg. Chamaecerasus is characterized by two-flowered cymes and free leaves, whereas subg. Lonicera has three-flowered cymes in whorls and perfoliate leaves subtending the inflorescences. Subg. Chamaecerasus, consisted of 53 species, was further divided into four sections, i.e., sect. Coeloxylosteum, sect. Isika, sect. Isoxylosteum and sect. Nintooa, based mainly on habit, pith, bracteole, corolla and ovary. The most specious one, sect. Isika, consisting of 35 species, was divided into eight subsections. Among them, subsect. Alpigenae is defined by the combination of shrubs deciduous, corolla bilabiate, corolla lobes longer than tube which is shallowly gibbous toward base, style and the inner side of the gibbous densely villous. Three species were recognized in this subsection, i.e., *L.fargesii*, *L.oblata* and *L.webbiana*. A number of molecular phylogenetic studies have been conducted for *Lonicera*, while sect. Isika was recovered as polyphyletic, most subsections were proved to be monophyletic (Srivastav et al. 2023; Sun et al. 2023; Yang et al. 2024).

During our fieldwork in Lanzhou City, Gansu Province, China, several interesting specimens of *Lonicera* were collected. The leaf shape of these individuals was distinctly different from those of the described species of *Lonicera*. After careful morphological comparisons and literature consulting, we found that these specimens should be a new species belonging to subsect. Alpigenae of sect. Isika of subg. Chamaecerasus and morphologically similar to *L.webbiana*. The genetic differentiation and phylogenetic analysis using nuclear ribosomal internal transcribed spacer (ITS) and the combined sequences of chloroplast *psb*A-*trn*H and *mat*K fragments supported these specimens as a separate species. In this study, we name it as *Loniceralanzhouensis* Shun Liu & Yu J. Wang.

## ﻿Material and methods

Nine individuals of the new species were collected from Lanzhou City of Gansu province. At the same time, seven individuals of *L.webbiana*, morphologically resembling the new species and inhabiting similar altitude, was sampled and careful morphological comparison between the two species was made focusing especially on leaves, bract, bracteole, fruit and corolla in different periods. For phylogenetic analysis, both species, and additionally, 24 species representative of different sections of *Lonicera* were sampled. In total, 40 samples of ingroups and one sample of outgroup, according to the recent phylogenetic studies (Fan et al. 2018; Wang et al. 2021), were sampled and the accession number and collection information are shown in Table 1.

**Table 1.** The materials used for phylogenetic analyses of *Loniceralanzhouensis*. All voucher specimen were sampled from China.

Taxon	Voucher specimen	Source	Genebank NO. (ITS, *psb*A-*trn*H, *mat*K)			Coordinate
*L.caerulea*	*Y. J. Wang WYJ20190402*6 (LZU)	Ruoergai, Sichuan	OM952618	OM987816	OM987816	33.445044°N,103.424365°E
*L.ferdinandi*	*Y. J. Wang WYJ201904069* (LZU)	Yongdeng, Gansu	OM952611	OM987809	OM987810	36.595757°N,102.789116°E
*L.gynochlamydea*	*Y. J. Wang WYJ201904014* (LZU)	Kangxian, Gansu	OM952647	OM987845	OM987846	33.362872°N,105.833188°E
*L.rupicola*	*Y. J. Wang WYJ201904047* (LZU)	Menyuan, Qinghai	OM952537	OM987735	OM987736	37.619121°N,101.321653°E
*L.fragrantissima*	*Y. J. Wang WYJ201904229* (LZU)	Fengxian, Shanxi	OM952656	OM987854	OM987856	34.244038°N,106.931784°E
*L.scabrida*	*Y. J. Wang WYJ201904193* (LZU)	Muli,Sichuan	OM952777	OM987975	OM987976	28.473993°N,100.531588°E
*L.hispida*	*Y. J. Wang WYJ201904124* (LZU)	Ruoergai, Sichuan	OM952778	OM987976	OM987977	33.445044°N,103.424365°E
*L.stephanocarpa*	*Y. J. Wang WYJ201904128* (LZU)	Liuba, Shanxi	OM952792	OM987990	OM987991	33.693231°N,106.696817°E
*L.litangensis*	*Y. J. Wang WYJ201904157* (LZU)	Songpan, Sichuan	OM952794	OM987992	OM987993	32.443073°N,103.453454°E
*L.microphylla*	*Y. J. Wang WYJ201904094* (LZU)	Tianzhu, Gansu	OM952661	OM987887	OM987887	36.701042°N,102.748843°E
*L.tangutica*	*Y. J. Wang WYJ201904004* (LZU)	Ruoergai, Sichuan	OM952669	OM987857	OM987858	33.192257°N,103.452894°E
*L.acuminata*	*Y. J. Wang WYJ201904037* (LZU)	Shimian, Sichuan	OM952571	OM987769	OM987770	29.172155°N,102.244466°E
*L.japonica*	*Y. J. Wang WYJ201904064* (LZU)	Liuba, Shanxi	OM952606	OM987804	OM987805	33.535809°N,106.971391°E
*L.similis*	*Y. J. Wang WYJ201904060* (LZU)	Huixian, Gansu	OM952563	OM987761	OM987762	33.609354°N, 106.135348°E
*L.lanceolata*	*Y. J. Wang WYJ201904018* (LZU)	Wenchuan, Sichuan	OM952747	OM987945	OM987946	31.303924°N,103.466311°E
*L.nervosa*	*Y. J. Wang WYJ201904109* (LZU)	Tianzhu, Gansu	OM952724	OM987922	OM987923	36.692745°N,102.699308°E
*L.retusa*	*Y. J. Wang WYJ201904098* (LZU)	Fengxian, Shanxi	OM952697	OM987895	OM987896	34.062340°N,106.849433°E
*L.chrysantha*	*Y. J. Wang WYJ201904104* (LZU)	Tianzhu, Gansu	OM952708	OM987906	OM987907	36.714637°N,102.720511°E
*L.crassifolia*	*Y. J. Wang WYJ201904009* (LZU)	Jiulong, Sichuan	OM952773	OM987971	OM987972	29.125733°N,102.056948°E
*L.trichosantha*	*Y. J. Wang WYJ201904008* (LZU)	Hanyuan, Sichuan	OM952759	OM987957	OM987958	29.263633°N,102.501835°E
*L.maackii*	*Y. J. Wang WYJ201904006* (LZU)	Wenxian, Gansu	OM952738	OM987936	OM987937	32.523705°N,104.373583°E
*L.ruprechtiana*	*Y. J. Wang WYJ201904113* (LZU)	Yi’an, Heilongjiang	OM952734	OM987932	OM987933	47.912548°N,125.305214°E
*L.tragophylla*	*Y. J. Wang WYJ201904039* (LZU)	Fengxian, Shanxi	OM952529	OM987727	OM987728	34.244038°N,106.931784°E
*L.ligustrina*	*Y. J. Wang WYJ201904003* (LZU)	Shimian, Sichuan	OM952632	OM987830	OM987831	29.190707°N,102.254878°E
*L.webbiana*	*Y. J. Wang WYJ201904100* (LZU)	Ruoergai, Sichuan	OM952698	OM987896	OM987897	33.192257°N,103.452894°E
*L.webbiana*	*Y. J. Wang WYJ201904101* (LZU)	Lanzhou, Gansu	OM952699	OM987897	OM987898	35.885937°N,103.903642°E
*L.webbiana*	*Y. J. Wang WYJ201904102* (LZU)	Lanzhou, Gansu	OM952700	OM987898	OM987899	35.898251°N,103.896021°E
*L.webbiana*	*Y. J. Wang WYJ201904272* (LZU)	Lanzhou, Gansu	OM952701	OM987899	OM987900	35.915682°N,103.907493°E
*L.webbiana*	*Y. J. Wang WYJ201904273* (LZU)	Lanzhou, Gansu	OM952702	OM987900	OM987901	35.915682°N,103.907493°E
*L.webbiana*	*Y. J. Wang WYJ201904274* (LZU)	Lanzhou, Gansu	OM952703	OM987901	OM987902	35.915682°N,103.907494°E
*L.webbiana*	*Y. J. Wang WYJ201904277* (LZU)	Lanzhou, Gansu	OM952704	OM987902	OM987903	35.915682°N,103.907495°E
*L.lanzhouensis*	*Y. J. Wang WYJ201904106* (LZU)	Lanzhou, Gansu	OM952715	OM987913	OM987914	36.719479°N,102.620805°E
*L.lanzhouensis*	*Y. J. Wang WYJ201904107* (LZU)	Lanzhou, Gansu	OM952716	OM987914	OM987915	36.712555°N,102.631604°E
*L.lanzhouensis*	*Y. J. Wang WYJ201904108* (LZU)	Lanzhou, Gansu	OM952717	OM987915	OM987916	36.810001°N,102.994549°E
*L.lanzhouensis*	*Y. J. Wang WYJ201904287* (LZU)	Lanzhou, Gansu	OM952718	OM987916	OM987917	36.726978°N,102.603234°E
*L.lanzhouensis*	*Y. J. Wang WYJ201904288* (LZU)	Lanzhou, Gansu	OM952719	OM987917	OM987918	36.727188°N,102.605435°E
*L.lanzhouensis*	*Y. J. Wang WYJ201904289* (LZU)	Lanzhou, Gansu	OM952720	OM987918	OM987919	36.7192°N,102.621335°E
*L.lanzhouensis*	*Y. J. Wang WYJ201904290* (LZU)	Lanzhou, Gansu	OM952721	OM987919	OM987920	36.710396°N,102.634037°E
*L.lanzhouensis*	*Y. J. Wang WYJ201904291* (LZU)	Lanzhou, Gansu	OM952722	OM987920	OM987921	36.707924°N,102.635953°E
*L.lanzhouensis*	*Y. J. Wang WYJ201904292* (LZU)	Lanzhou, Gansu	OM952723	OM987921	OM987922	36.707694°N,102.698808°E
*Symphoricarpossinensis*	*Y. J. Wang WYJ201904158* (LZU)	Wenxian, Gansu	OM952564	KP297634	MK136238	32.518879°N,104.445264°E

Total DNA was extracted from fresh leaves using the modified CTAB method (Doyle and Dickson 1987). The nuclear ribosome ITS and the chloroplast *psb*A-*trn*H and *mat*K fragments were sequenced and submitted to NCBI following Chen and Wang (2018). We concatenated the two chloroplast fragments into one matrix and two datasets, i.e., ITS and chloroplast (CP) concatenated sequences, were used in the following analyses. MEGA 11.0.13 (Tamura et al. 2021) was used to align the two datasets and calculate the genetic distances. MrBayes 3.1.2 (Huelsenbeck and Ronquist 2001) was used to make Bayesian Inference (BI) under the GTR+G model, with 4 chains run at the same time, the temperature and other parameters of the chain took the default value. The analysis was performed 10 million times, sampling was taken every 1000 generations. The convergence of the data was detected by Tracer1.7 (Rambaut et al. 2018), which ensured that the ESS > efficiently sampled 200. The first 25% of the trees were discarded as burn-in, whereas the remaining trees were used to calculate the 50% majority consensus tree using PAUP4.0 (Swofford 2002).

## ﻿Results

**Figure 1.**
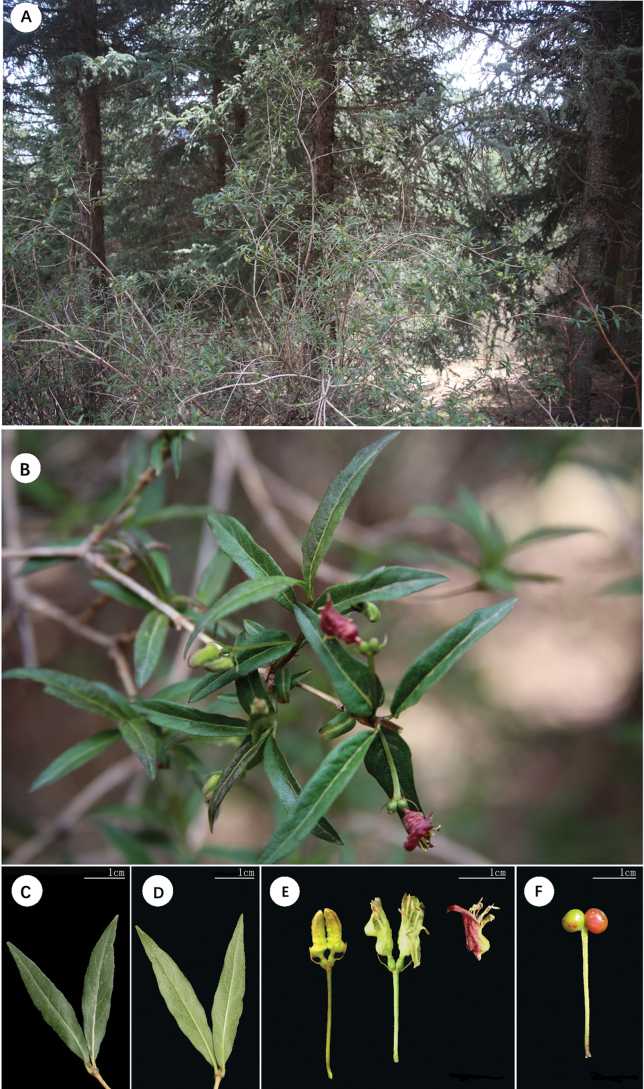
*Loniceralanzhouensis***A** habitat **B** whole individual **C** adaxial surface of leaf **D** abaxial surface of leaf **E** corolla at different stages **F** fruit.

**Figure 2.**
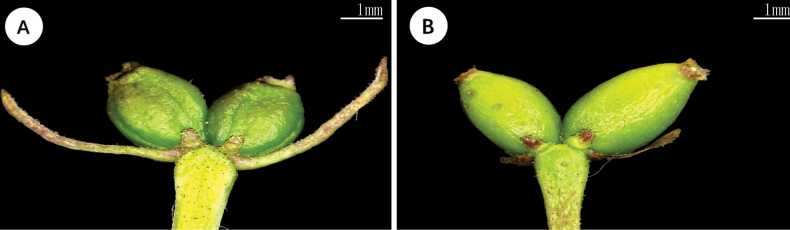
The comparison of involucral bracts between *L.lanzhouensis* (Left) and *L.webbiana* (Right).

**Figure 3.**
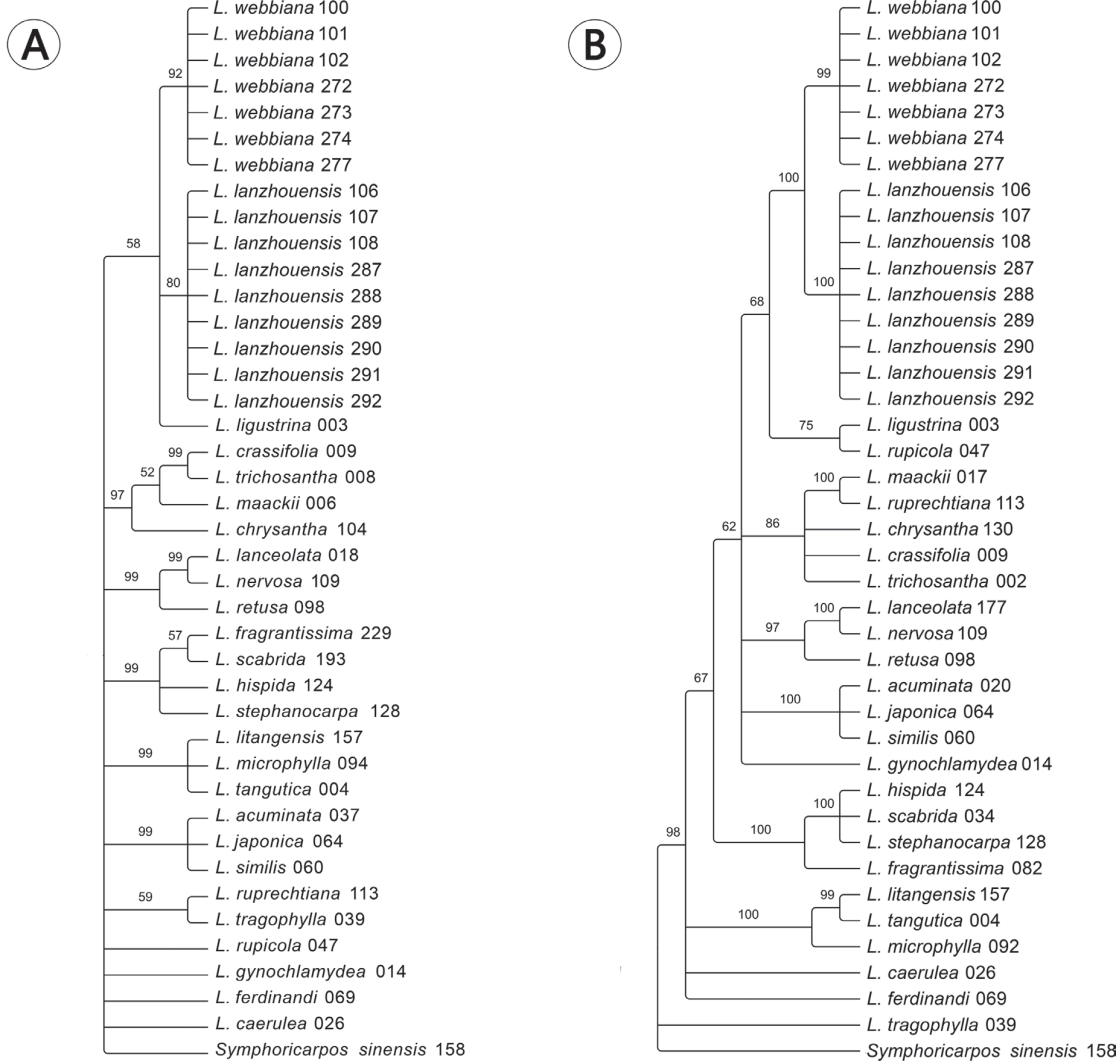
Bayesian Inference (BI) based on ITS (**A**) and the concatenated sequences of *psb*A-*trn*H and *mat*K (**B**). Posterior probabilities (PP) are indicated above branches. The digits following the species name represent the last three digits of the specimen number.

Besides the conspicuous difference in the shape of leaf, we further found that the new species differentiated from *L.webbiana* in the shape of bract. At the same time, the color of corolla varied in different periods of blossoms for the new species, but that of *L.webbiana* remained purple-red for most time (Figs 1, 2). The aligned ITS sequence matrix contained 742, of which 97 were varied and 39 were parsimony informative. The average distance among the ingroups is 2.08%, and ranged from 0 (between *L.crassifolia* and *L.trichosantha*) to 5.4% (between *L.japonica* and *L.trichosantha* or *L.crassifolia*). No intraspecies distance was detected within the new species or *L.webbiana* and the inter-species distance between the two species is 0.27%, identical to that between *L.lanceolata* and *L.nervosa*. The phylogenetic analysis recovered 11 well supported clades (Fig. 3), including one composed of the new species, *L.webbiana* (PP = 58) and *L.ligustrina*, but the relationship among the clades is unclear. The aligned CP sequence contained 1335 bp, of which 92 are varied and 46 are parsimony informative. The average distance among the ingroups is 1.46%. The distances between *L.hispida* and *L.stephanocarpa* (0), *L.crassifolia* and *L.trichosantha* (0.01%), *L.lanceolata* and *L.nervosa* (0.01%), *L.crassifolia* and *L.chrysantha* (0.3%) are among the lowest and the counterpart of ITS are 1.1%, 0, 0, 0.27%, respectively. The distance between *L.litangensis* and *L.retusa* (3%) is the largest with a counterpart of ITS 2.8%. The inter-species distance between the new species and *L.webbiana* is 0.72% and additionally, a deletion of 7-base pairs was detected in the new species. The intraspecies distance within *L.webbiana* was 0.02% while that within the new species is 0. The results (Fig. 3) from phylogenetic analysis are slightly different from that of ITS, such as the division of two major clades corresponding to the two subgenera. But the close relationship between the new species and *L.webbiana* is similarly supported in that they formed a subclade (PP = 100) sister to the subclade composed of *L.ligustrina* from sect. Isika and *L.rupicola* from sect. Isoxylosteum (PP = 68).

## ﻿Discussion

On the basis of erect shrub, bilabiate flower with short tube gibbous on ventral side toward base, the new specie could be easily assigned to subsect. Alpigenae of sect. Isika of subg. Chamaecerasus. Phylogenetic analysis from both CP and ITS revealed that the new species and *L.webbiana*, a member of subsect. Alpigenae, formed a monophyletic clade within *Lonicera*. The genetic distances between the new species and *L.webbiana*, are 0.27% based on ITS or 0.72% based on CP dataset. Such a low distance, on the one hand, supported their similarity in morphology and on the other hand, imply the scenario to treat them as two varieties. But we prefer the treatment of two species in that 1) their distance based on ITS are larger than *L.crassifolia* and *L.trichosantha*, from sect. Nintooa and sect. Coeloxylosteum, respectively, 2) their distance based on CP is significant and larger than many species-pairs; 3) the morphological difference in the length of bract, the shape of leaf and the color of corolla is distinct. Therefore, both morphology and molecular evidences, including CP and ITS, are consistent and support the closely relationship between the new species and *L.webbiana*, and at the same time, their distinction.

### ﻿Taxonomic treatment

#### ﻿ Lonicera lanzhouensis

Taxon classificationPlantaeDipsacalesCaprifoliaceae

Shun Liu & Yu J. Wang sp. nov.

2EC807E5-DA73-5213-A76F-7D8CF06E67A5

urn:lsid:ipni.org:names:77362013-1

Figs 1 , 2

##### Type.

China • Gansu: Lanzhou, 36.810001°N, 102.994549°E, 3012 m elev., May thirtieth, 2022, Y. J. Wang *WYJ201904293* (Holotype, LZU!; Isotypes, LZU!), Figs 1, 2.

##### Description.

Shrub erect, up to 2 m tall. Stems cylindrical, branched. Branches solid with white pith. Petiole 1.5–2.5 cm long, inconspicuously pubescent. Leaves opposite, leaf blade narrowly lanceolate, stiffly papery, 2–4 cm × 0.5–0.8 cm, hairy, narrow, margin with irregular undulations, entire, apex tapering. Inflorescence thyrsoid, axillary, cymes opposite and reduced to paired flowers, pedunculate with a pair of bracts and 2 pairs of bracteoles; bracts long, ca. 4.5 mm × 0.5 mm, bracteoles wide and short, ca. 0.5 mm × 0.5 mm. Paired flowers with free ovaries. Pedicels 1.5–2.5 cm long, Calyx 5-lobed, with sparse glandular hair. Corolla bilabiate, yellow-green at the beginning, turns into reddish-purple after anthesis, ca. 1.4 × 0.5 cm; tube shallowly to deeply gibbous toward base, outside sparsely spreading hairy, inside puberulent; lower lip slightly recurved; upper lip 4-lobed to middle. Stamens subequaling corolla, filaments pubescent. Ovary inferior, locules 2; style ca. 1.1 cm long, slender, hairy; stigmas capitate. Fruit a berry, round, green, turns red when mature, ca. 0.6 cm in diameter.

*L.lanzhouensis* is morphologically most similar to *L.webbiana* and can be readily distinguished from it by having narrow leaves, yellow-green flowers at the beginning and reddish-purple when mature, and long involucrate bracts. In contrast, *L.webbiana* has ovate-elliptic to ovate-lanceolate leaves, red flowers and short involucrate bracts.

##### Phenology.

Flowering: May to June, Fruiting: June.

##### Distribution and habitat.

*L.lanzhouensis* is found only in Lanzhou, Gansu, China with a population of about 500 individuals. It grows in a spruce forest at elevation of 2870–3300 m.

##### Additional specimens examined.

China • Gansu: Lanzhou City, Yongdeng County, 36.719479°N, 102.620805°E, 3271 m elev., June 27^th^, 2018, Y. J. Wang *WYJ201904106* (LZU!); • 36.712555°N, 102.631604°E, 3254 m elev., June 27^th^, 2018, Y. J. Wang *WYJ201904107* (LZU!); • 36.810001°N, 102.994549°E, 3012 m elev., July 1^st^, 2018, Y. J. Wang *WYJ201904108* (LZU!); • 36.726978°N, 102.603234°E, 3300 m elev., July 12^th^, 2018, Y. J. Wang *WYJ201904287* (LZU!); • 36.727188°N, 102.605435°E, 3211 m elev., July 12^th^, 2018, Y. J. Wang *WYJ201904288* (LZU!); • 36.7192°N, 102.621335°E, 3005 m elev., July 13^th^, 2018, Y. J. Wang *WYJ201904289* (LZU!); • 36.710396°N, 102.634037°E, 2907 m elev., July 13^th^, 2018, Y. J. Wang *WYJ201904290* (LZU!); • 36.707924°N, 102.635953°E, 2878 m elev., July 13^th^, 2018, Y. J. Wang *WYJ201904291* (LZU!); • 36.707694°N,102.698808°E, 3297 m elev., August 19^th^, 2019, Y. J. Wang *WYJ201904292* (LZU!).

##### Chinese name.

Lanzhou rendong (兰州忍冬).

##### Etymology.

The specific epithet indicates its type locality, Lanzhou City, Gansu Province, China.

## Supplementary Material

XML Treatment for Lonicera lanzhouensis

## ﻿Additional information

### Author ORCIDs

Yu-Jin Wang https://orcid.org/0000-0001-7448-7526

### Data availability

All of the data that support the findings of this study are available in the main text.
